# The role of glutamine synthetase isozymes in enhancing nitrogen use efficiency of N-efficient winter wheat

**DOI:** 10.1038/s41598-017-01071-1

**Published:** 2017-04-20

**Authors:** Zhiyong Zhang, Shuping Xiong, Yihao Wei, Xiaodan Meng, Xiaochun Wang, Xinming Ma

**Affiliations:** 1 Collaborative Innovation Center of Henan Grain Crops, Henan Agricultural University, Zhengzhou, 450002 China; 2 College of Agronomy, Henan Agricultural University, Zhengzhou, 450002 China; 3 Key Laboratory of Physiology, Ecology and Genetic Improvement of Food Crops in HenanProvince, Zhengzhou, 450002 China; 4 Department of Biochemistry, College of Life Science, Henan Agriculture University, Zhengzhou, 450002 China

## Abstract

Glutamine synthetase (GS) isozymes play critical roles in nitrogen (N) metabolism. However, the exact relationship between GS and nitrogen use efficiency (NUE) remain unclear. We have selected and compared two wheat cultivars, YM49 and XN509, which were identified as the N-efficient and N-inefficient genotypes, respectively. In this study, agronomical, morphological, physiological and biochemical approaches were performed. The results showed that *TaGS1* was high expressed post-anthesis, and *TaGS2* was highly expressed pre-anthesis in N-efficient genotype compared to N-inefficient genotype. GS1 and GS2 isozymes were also separated by native-PAGE and found that the spatial and temporal distribution of GS isozymes, their expression of gene and protein subunits in source-sink-flow organs during development periods triggered the pool strength and influenced the N flow. According to the physiological role of GS isozymes, we illustrated four metabolic regulation points, by which acting collaboratively in different organs, accelerating the transport of nutrients to the grain. It suggested that the regulation of GS isozymes may promote flow strength and enhance NUE by a complex C-N metabolic mechanism. The relative activity or amount of GS1 and GS2 isozymes could be a potential marker to predict and select wheat genotypes with enhanced NUE.

## Introduction

Nitrogen (N) is one of the major crop nutrients applied to fields to improve yields^1^. Although increasing amounts of commercial fertilisers are applied in developing countries, the amount of N in some fertilisers actually used by crops is limited^2^, which not only wastes resources but also leads to serious environmental damage through ground and surface water pollution caused by nitrate (NO_3_^−^) leaching^3, 4^. Nitrogen use efficiency (NUE), which is defined as the grain yield per unit of available N from the soil^5^, is considered to be only 30–40% for winter wheat in the North China Plain^6^. Most of the N added to the soil is lost to the environment. Given these circumstances, it is important to identify the limiting steps in N metabolism, including N uptake, assimilation, remobilisation, and storage^7^, to optimise the use of N which is vital to the sustainability of agriculture.

Plant N management involves two main steps: uptake and utilisation. In wheat, nitrate was mainly absorbed by roots, which is reduced to nitrite by nitrate reductase (NR)^8^. NR catalyzes the reduction of nitrate to nitrite with pyridine nucleotide in N assimilation in higher plants^9^. Since nitrite is highly reactive, plant cells transport them into chloroplasts of the green photosynthetic tissues by vascular bundles. In these green organs, nitrite is further reduced to NH_4_^+^ by nitrite reductase (NiR) for assimilation. The enzymes mainly involved in nitrogen assimilation in plants are glutamine synthetase (GS), glutamate synthase (GOGAT) and glutamate dehydrogenase (GDH). But their effects are not equivalent, in which the GDH has a relatively low affinity (Km = 8–10 mM) for ammonium than that of GS (Km ≈ 0.1 mM)^10^. The role of GDH is to catalyse glutamate releasing ammonium for regulating the C/N ratio^11^ and assimilate ammonium for preventing ammonium poisoning under stress conditions^12^. The GS/GOGAT cycle is the main pathway of ammonium assimilation in higher plants, and approximately 95% of the NH_4_^+^ assimilation via this cycle^13, 14^. Once N has been taken up and assimilated, it is transported throughout the plant, predominantly in the form of glutamine, asparagine, glutamate and other nitrogenous compounds, for utilisation and storage^15^. The most abundant protein in plants is ribulose-1-5 bis-phosphate carboxylase-oxygenase (Rubisco) (EC 4.1.1.39), which is the fundamental enzyme of plant photosynthesis, accounting for more than 60% of leaf soluble protein, and is believed to constitute the main N reserve in the vegetative tissues of wheat^16^.

Glutamine synthetase (GS) (EC 6.3.1.2) is a key enzyme in the first step of NH_4_^+^ assimilation, which is responsible for the synthesis of glutamine. GS isozymes have different metabolic roles, and their activities vary during plant development in different organs and cell types. Two GS isozymes, GS1 and GS2, are present in higher plants^17^. GS1 is usually found in the cytosol of vascular tissues and encoded by 3–5 nuclear genes^18^. It is involved primarily in N recycling in senescent leaves and N translocation during seed germination^19, 20^. GS2 is found primarily in the plastids of green tissues and encoded by a single nuclear gene^21^. It is mainly involved in the photorespiratory release of NH_3_, and in NO_3_^−^ reduction during NH_3_ assimilation^22, 23^. Given that the released NH_3_ by photorespiration is 10-fold higher than that of NO_3_^−^ reduction; the activity of GS2 plays an important role in the efficiency of N assimilation by preventing N loss.

GS isozymes are regulated differently in the main crop species^24–26^. In maize (*Zea mays* L.), the GS1-4 mutant was characterised by reduced kernel size, and the GS1-3 mutant exhibited reduced kernel number. The aboveground biomass, however, remained almost the same, showing that GS1 plays a major role in controlling N transfer and recycling^27^. Overexpression of the GS1 gene can improve crop production in rice^28^. Haplotype analysis revealed the GS2 gene location and four major *TaGS2* haplotypes (A1b, B1a, B1b, D1a) of 266 wheat cultivars, which may confer with a better N use and agronomic traits under differing N conditions^29^. Genetic studies in durum wheat and rice localised the GS genes using quantitative trait locus (QTL) analysis for grain protein content (GPC), cytosolic glutamine synthetase content, and panicle number^30, 31^. Increased GS expression in transgenic rice also made the rice tolerant to N-deficiency^32^. Principal component analysis (PCA) and correlation studies performed in several cereal species suggested the presence of a strong relationship between GS activity and total N, chlorophyll, soluble protein, NH_4_^+^, and amino acids^33, 34^.

The literature is rather scarce in terms of two typical genotypes of different NUE. Moreover, GS isozymes have non-overlapping roles in N metabolism in many plant species, but in most previous studies, GS1 and GS2 isozymes were analysed together without separation of their individual activities. The mechanisms underlying how GS isozymes enable the supply of different levels of N to influence NUE in various wheat organs are unclear. To investigate the influence of GS isozymes on NUE, two representative wheat genotypes with different NUE were used. We provided a potential mechanism of GS and aimed to assess the factors that influence NUE by examining the N metabolism indicators, the activities, transcripts and subunit amounts in spatial and temporal distribution of GS isozymes. Based on the metabolic indicators and GS profiles, combining with their physiological roles, a schematic diagram was described to explain mechanisms leading to the NUE differences of two genotypes.

## Results

### Phenotypic and Agronomic Traits of Two Wheat Genotypes

**Figure 1:**
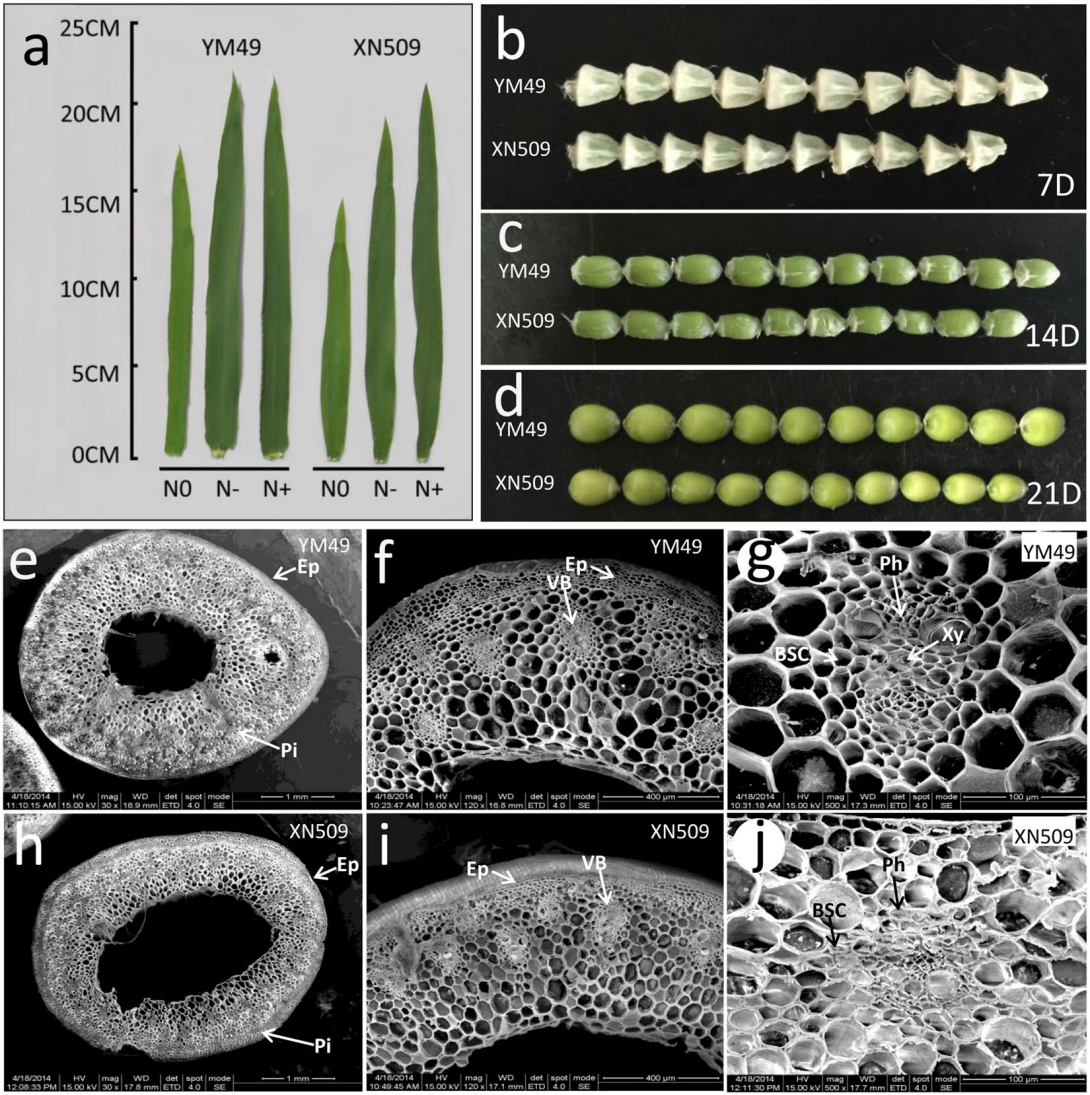
Phenotypic traits of the two wheat genotypes. (**a**) Leaf morphology of two wheat genotypes under various N conditions at the booting stage. (**b**) Kernel morphology of two wheat genotypes at 7D. (**c**) Kernel morphology of two wheat genotypes at 14D. (**d**) Kernel morphology of two wheat genotypes at 21D. (**e**–**g**) Morphological and structural observations of a stem section of YM49. (**e**) Overview of the Stem of YM49. (**f**) Overview of the Stem of YM49 containing a lateral vein. (**g**) Magnification of the vascular bundle (VB), showing the presence of bundle sheath cells (BSC), xylem (Xy) and phloem (Ph) of YM49. (**h**–**j**) Morphological and structural observations of a stem section of XN509. (**h**) Overview of the Stem of XN509. (**i**) Overview of the Stem of XN509 containing a lateral vein. (**j**) Magnification of the vascular bundle (VB), showing the presence of bundle sheath cells (BSC), xylem (Xy) and phloem (Ph) of XN509. Xy, xylem; Ph, phloem; BSC, bundle sheath cells; VB, vascular bundle; Pi, pith; Ep, epidermis. Note: Leaf was chosen from the flag leaves of the main stem at Feekes 10.0; kernel was chosen form the middle grain of the second spikelet for taking photos; the stem was chosen from the base of the second internode of the main stem.

The two wheat genotypes exhibited morphological differences in organs (Fig. 1). The functional leaves of the YM49 (N-efficient genotype) had a larger photosynthetic area than the XN509 (N-inefficient genotype) under different N conditions, which affected not only the leaf length and color (Fig. 1a), but also the leaf area index (LAI) (Fig. S2c). For example, the leaf lengths of YM49 (22.2 cm) were 17.46% higher than that of XN509 (18.9 cm) under N− level. According to the chlorophyll content under three N conditions (Fig. S2a), YM49 showed a greater stay-green ability than XN509 after anthesis. The net photosynthetic rate (Pn) of YM49 was 7.80% higher than XN509 at AS under N− conditions, implying YM49 has stronger assimilation ability (Fig. S2b).

Moreover, the ear number per plant and grain number per ear were also changed with the N level. The more N was applied, the more grain and ear number the plant had, what’s more, there were no much differences between two cultivars under the same N level. The 1000-grain weight of YM49 was about 48 g and XN509 was about 40 g, and the kernels of YM49 developed more plump during grain filling stages (Fig. 1b–d). Compared with N+, the yield of YM49 per plant decreased 0.03 g, while that of XN509 decrease 0.62 g under N− (Table S5). The grain filling rates of YM49 (0.5–2.1 mg per grain per day) were 20% faster than that of XN509 (0.4–1.8 mg per grain per day) during grain filling stage (Fig. S3a). Specifically, the grain filling rates of the N-efficient genotype increased faster than the N-inefficient genotype from 7 days after anthesis (7D) to 14 days after anthesis (14D). The grain of YM49 were phenotypically larger than XN509 (Fig. 1b), which means YM49 owned a bigger storage capacity in sink organs, and dry matter accumulation data also showed that YM49 owned a bigger storage capacity under the plant level (Fig. S3b).

In wheat, the stem plays an important role in nutrient translocation and remobilisation from the vegetative organs to the sink organs. The SEM observations of stem (Fig. 1e–j) showed that the stem wall of YM49 was 37.61% thicker than that of XN509 (Table S2). YM49 also had more and greater vascular bundles than XN509. The phloem area exhibited a similar trend, suggesting that the transport channel of nutrient in YM49 was more fluent.

### Nitrogen Metabolic Features of Two Wheat Genotypes

**Figure 2:**
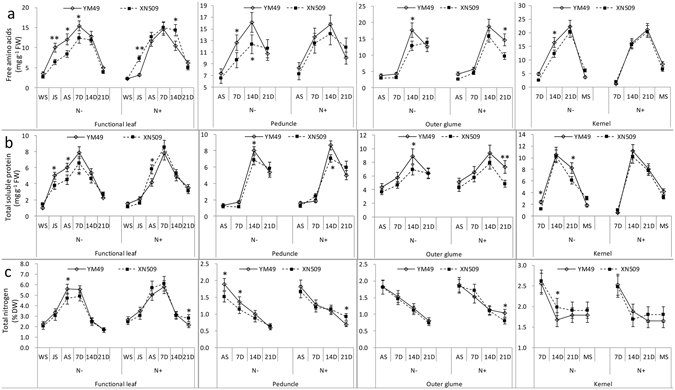
Changes in the indicators of N metabolism in relation to the plant growth stage and N level in various organs. Vertical bars correspond to the mean of three individual samples taken from three different pots ± SE. Asterisks (*p < 0.05, LSD) indicate significant difference between the two genotypes. Double asterisks (**p < 0.01, LSD) indicate extreme significant difference between the two genotypes.

Three N metabolism indicators, the content of free amino acid, soluble protein and total nitrogen in leaf, peduncle, outer glume and kernel were monitored from WS to MS under high (N+) and low (N−) N level (Fig. 2). The trend of developmental changes of three N metabolic indicators was similar between YM49 and XN509. The content of free amino acids, total soluble protein and total nitrogen were increased to the top from AS to 14D, and then decrease with the plant senescence. The difference of N metabolic indicators of XN509 was bigger under different N conditions, while that of YM49 were much smaller. In the functional leaf (the mainly assimilation organ), the free amino acid and soluble protein contents in YM49 were 30.53% and 16.91% higher, respectively, than in XN509 from AS to 7D under N−. Significant differences (*p* < 0.05) were observed of the contents of free amino acids and total soluble protein at JS in the functional leaf (Fig. 2a) between two wheat genotypes. In the peduncle (transport organ), significant differences were found at 7D or after. In the outer glume (assimilation organ and transport organ), the significant differences were found at 14D or 21D. An obvious decrease was observed since sampling from AS to MS of total nitrogen in peduncle, outer glume and kernel (Fig. 2c), but in the kernel remained at a constant level after 14D, which means the nitrogen compounds were are continuously transported to the grain form the source organs.

### GS Isozymes Activity and Relative Activity Analysis

**Figure 3:**
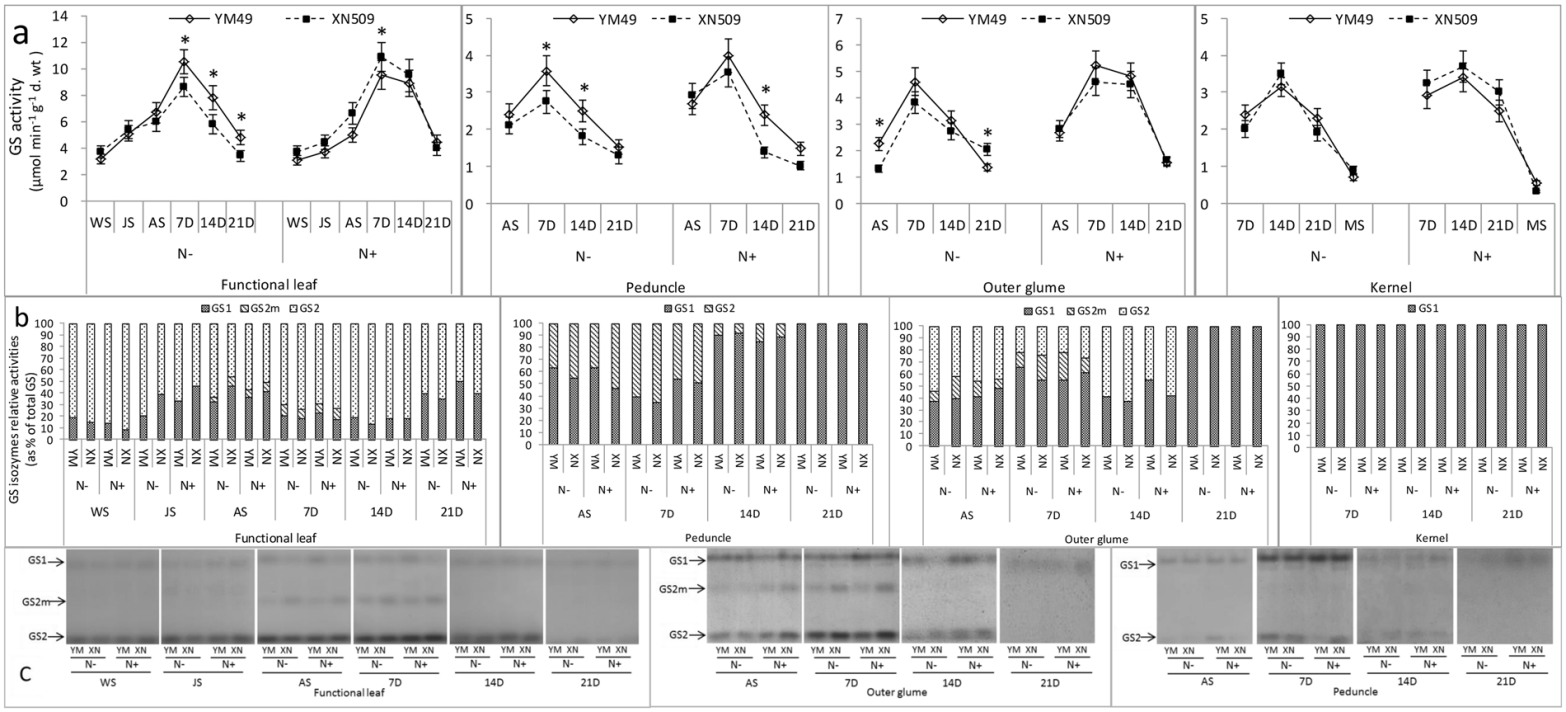
Glutamine synthetase (GS) activity and relative levels in various organs of two wheat genotypes. (**a**) GS activity. Data are the mean ± SE of three biological replicates. Asterisks (*p < 0.05, LSD) indicate significant differences between YM49 (YM) and XN509 (XN) under the same nitrate conditions. Low nitrogen (N−) and high nitrogen (N+). (**b**) Relative activities of GS1 and GS2 isozymes of two wheat genotypes. Proportions were calculated after quantification of signals using gray-scale image analysis and Gel-Pro Analyser software (Version 4.0). (**c**) GS1 and GS2 isozymes in different organs of two wheat genotypes were separated by native-PAGE. Equal protein amounts were loaded in each lane (50 μL).

Under both N− and N+ conditions, GS activity exhibited the same trend in both two genotypes. GS activity in the different organs was in the following order: functional leaf > outer glume > peduncle > kernel. GS activity peaked at 7D in the functional leaf, while the highest activity was observed at 14D in the kernel. In the functional leaves and outer glume, GS maintained a higher activity under N+ but decreased sharply under N− at 14D. In the functional leaf, peduncle and the outer glume, the GS activity of YM49 was higher than that of XN509. Significant differences were noticed at 7D, 14D and 21D in the functional leaves. GS activity in the peduncle was much higher in YM49 than in XN509 during the entire post-anthesis periods (Fig. 3a). In the kernel, however, GS activity was almost identical and no significant difference was observed between the two genotypes.

Native-PAGE combined with grey-scale image analysis enabled to estimate the relative quantification of the GS1 and GS2 isoforms, and calculate their respective proportions (Fig. 3b). Compared with GS2, the activity of GS1 was low during the vegetative growth periods (from WS to AS) in the source organs (leaf and outer glume). The opposite was observed in the peduncle, and relative activities of GS1 and GS2 differed greatly during development. From AS to 7D, higher levels of GS2 were observed in the functional leaf and outer glume, and a third band (GS2m) was appeared in the functional leaf and outer glume. In the kernel only GS1 was found (Fig. S6), and the highest activity was appeared at 14D (Fig. 3a).

### Expression Analysis of GS Genes in Different Organs of Two Wheat Genotypes

**Figure 4:**
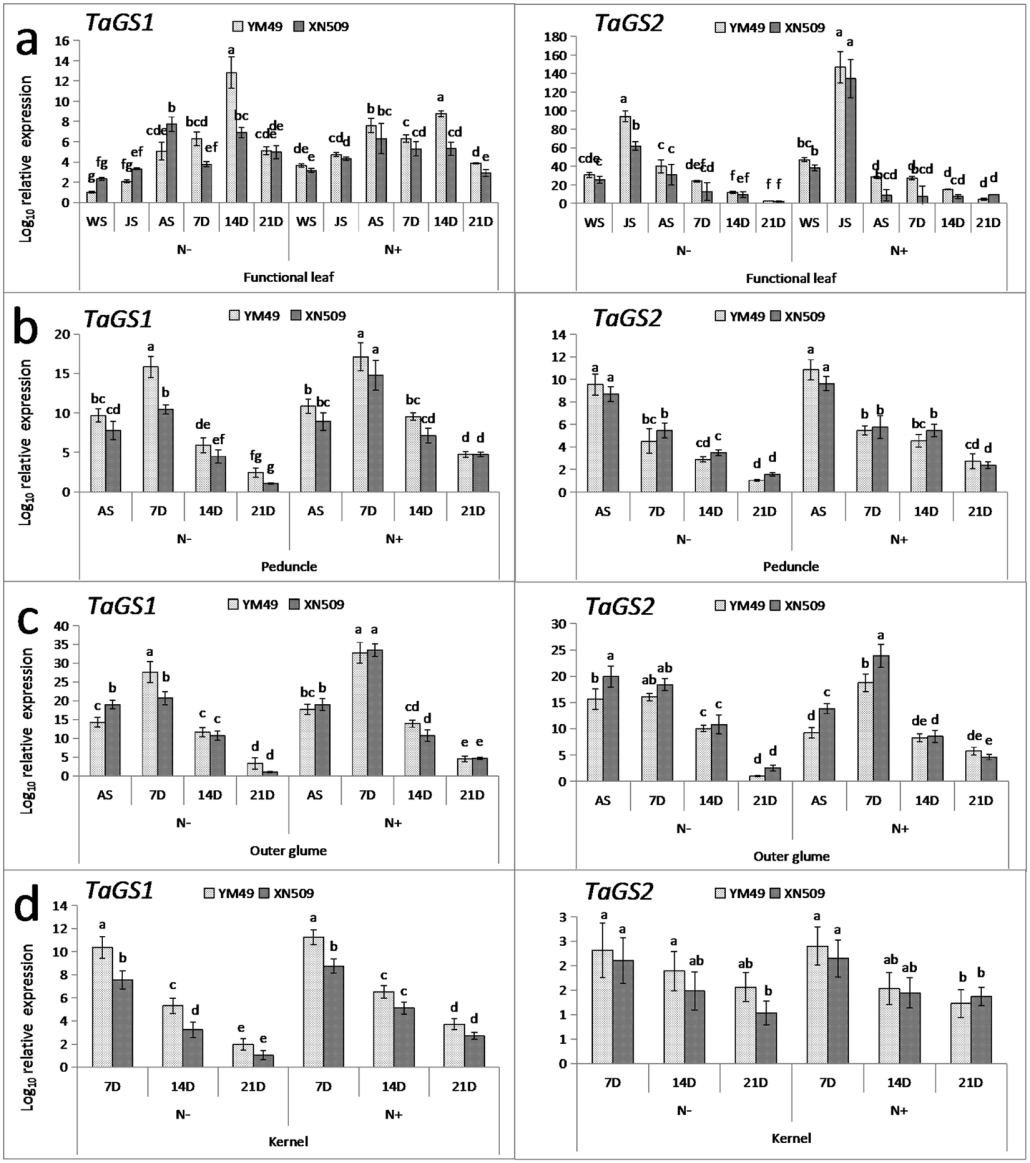
Transcription levels of the *TaGS1* and *TaGS2* genes in various plant organs grown under low (N−) and high (N+) nitrogen conditions. Log_10_ relative expression values are shown. Data are means ± SE (standard error), three biological replicates. Letters indicate statistical differences (*p* < 0.05). *TaGAPDH* was used as the reference gene.

The transcript level of *TaGS1* and *TaGS2* were monitored under low- or high-N conditions (Fig. 4). In the functional leaf, *TaGS1* expressed highly at 14D both under N− and N+, and showed the significant difference between two genotypes (Fig. 4a). *TaGS1* expression showed a similar pattern in the peduncle and outer glume, with the highest expression observed at 7D followed by a gradual decrease (Fig. 4b,c). At 7D, significant differences were also observed between YM49 and XN509 in all organs under N− level, but no significant difference was observed under N+ level. The major GS isoform in leaves, *TaGS2*, showed a prominent increase in expression with N increasing. The highest expression of *TaGS2* occurred at JS and differed significantly between YM49 and XN509, and its expression remained high compared to the *TaGS1* gene. After JS, *TaGS2* expression decreased rapidly under both N− and N+. In the peduncle, no significant difference was found during the whole stages. In the outer glume, *TaGS2* expression was significantly lower in YM49 than in XN509 at AS and 7D under N+. In the kernel, significant difference was found of *TaGS1* at 7D, but no difference observed of *TaGS2* during the whole grain filling stages (Fig. 4d). The *TaGS2* gene expression was almost tenfold than *TaGS1* gene in the functional leaf (Fig. 4a). The results indicated that the expression difference of *TaGS1* and *TaGS2* in different organs of critical stages may lead to the difference assimilation and translocation ability of two cultivars.

### Features of the Subunit content of GS Isozymes in Two Wheat Genotypes

**Figure 5:**
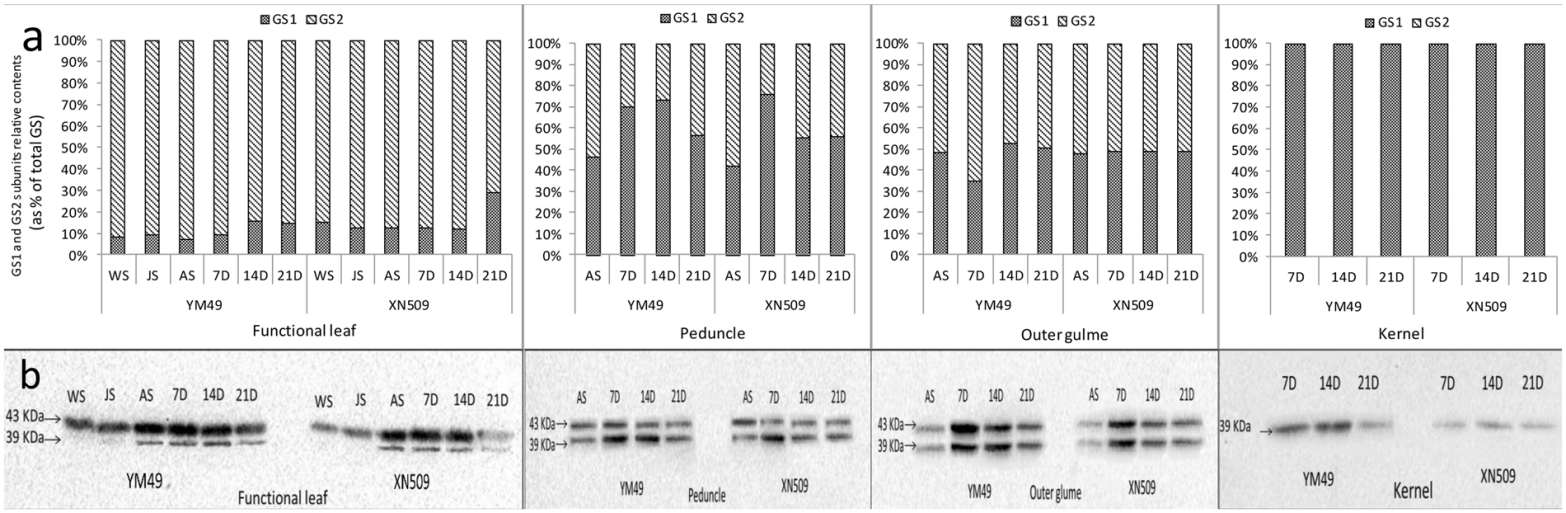
Western blot analysis of GS isozymes expression and dynamic changes in various organs under N− conditions. (**a**) Relative levels of GS1 and GS2 subunits; (**b**) Dynamic change in GS isozyme subunits in organs.

The dynamic changes of GS subunit levels in the two wheat genotypes were investigated by western-blot (Fig. 5b). In functional leaves, the GS2 subunits (42KDa) were more prevalent than GS1 (39KDa). GS2 subunit level peaked at 7D and then decreased. GS1 subunits level was low during the early growth stages, but increased after anthesis. YM49 exhibited higher GS2 subunits expression than XN509 during the WS and JS, and the expression of GS1 subunits in YM49 was also higher than in XN509 at 7D, 14D, and 21D. YM49 had a higher value of the ratio of GS2 subunit level to GS1 subunit level (GS2/GS1) than XN509 from WS to 7D in the functional leaf. In the peduncle, GS1 subunits were predominant, and the GS1 subunits were expressed more abundantly in YM49 than in XN509 from 7D to 14D (Fig. 5a). In the peduncle, the differences of the GS1/GS2 peaked at 14D, and the value in YM49 was 117.6% higher than in XN509 (Fig. 5a). In the outer glume, the value of GS1/GS2 was similar during the whole developmental stages. However, higher GS1 and GS2 subunit level was detected in YM49 at 7D and 14D (Fig. 5b). The subunit levels of GS isoforms were consistent with the transcripts of TaGS1 and TaGS2 (Fig. 4).

### Nitrogen Remobilisation Efficiency and N Contribution Rate to the Grains

There were significant differences of pre-anthesis N accumulation and post-anthesis N remobilisation between genotypes and N treatments (Table S7). The pre-anthesis N accumulation in YM49 was much higher than in XN509 under both N− and N+. The post-anthesis N remobilisation efficiency (NRE) and N contribution rate (NCR) to the grain differed significantly between the two genotypes under N−. The NRE in YM49 was 6.52% higher than that in XN509 under N− (Table S7). Under N+, no difference of NCR to the grain was observed in the two genotypes, and the mean of Pre- NCR of N+ was higher than that of N−.

### Schematic Diagram for GS Regulation of C/N Flux from Source to Sink

**Figure 6:**
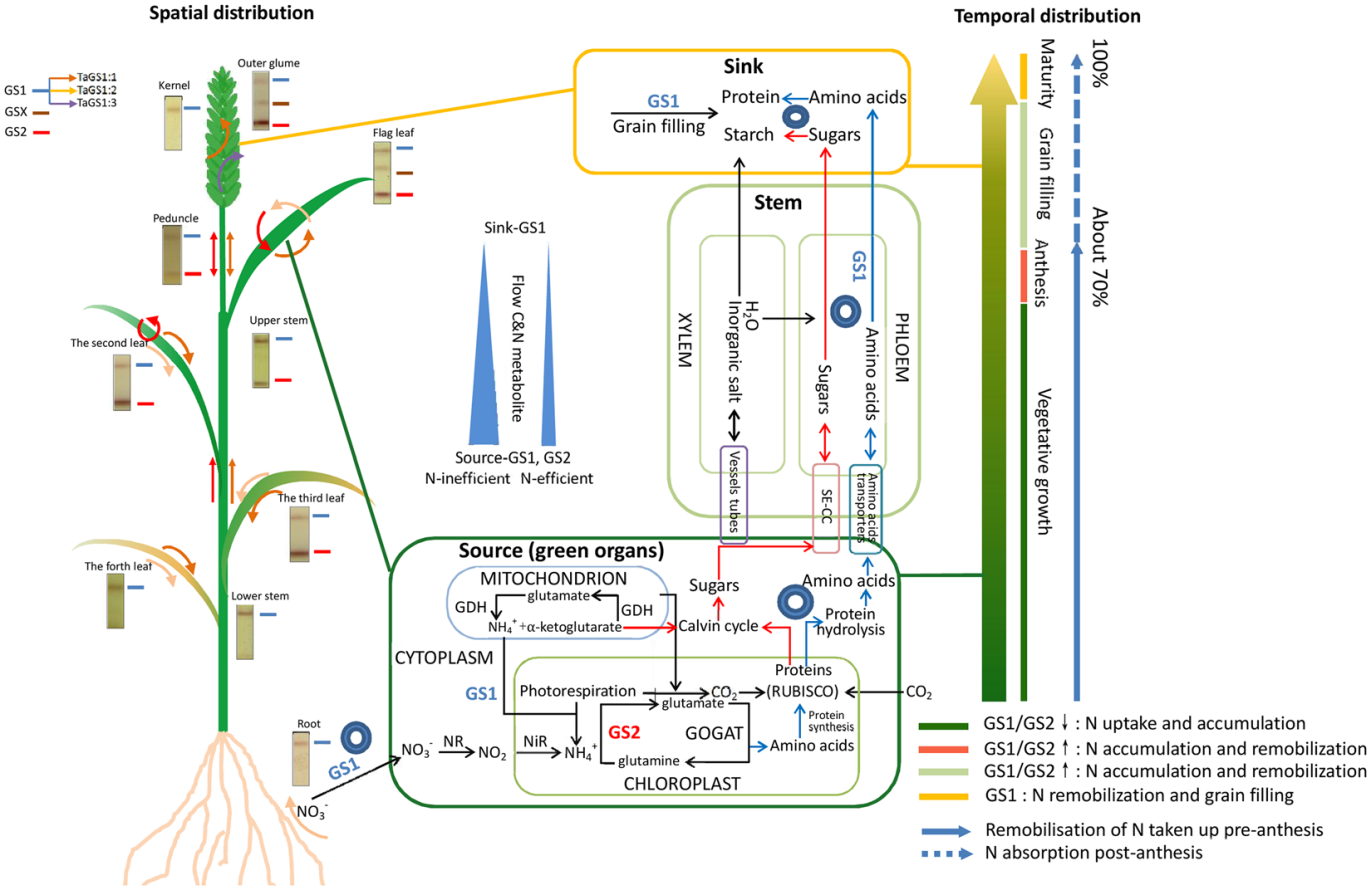
Schematic diagram of the spatial and temporal regulatory points of GS isozymes. Linear arrows indicate C and N fluxes. Blue arrows indicate amino acids fluxes. Red arrows indicate sugars fluxes. Concentric circles indicate the possible regulatory points through amino acid and sugar flow caused by pool strength in source-sink-flow organs.

GS isozymes are regulated at four main points, absorption in roots, assimilation in green organs, translocation through phloem and accumulation in grain (Fig. 6). During the vegetative stages, GS2 expression in N-inefficient genotype that is needed for regulation of N assimilation cannot meet the plant’s demand for photosynthetic protein synthesis and only a small amount of surplus protein is stored in the chloroplasts compared to the N-efficient genotype, although this does not affect the plant’s ability to sustain life, which results in less protein synthesis (i.e., RUBISCO). Carbon assimilation is also affected by protein synthesis, and the photosynthetic production of sugars in N-inefficient genotype were less than that in N-efficient genotype, resulting in sugars and amino acids in the export pool (source organs) being low during grain filling. Furthermore, GS1 isozymes play a role in the regulation of kernel number and size^27^. In the kernel, amino acids are used for protein synthesis and sugars are used for starch synthesis. The low capacities of kernels in N-inefficient wheat genotypes results in a highly import pool resistance, resulting in nutrients translocation to the kernels was confined. As a consequence, in the phloem, the N flux remains low between the source and the sink organs, which constrains the N translocation and remobilisation, and the most direct evidence is the grain filling rate (Fig. S3a). At maturity, the N was still remained in the stem or leaf, although it was absorbed by the plants roots. In the bundle sheath cells of the phloem, GS1 is likely to be involved in regulating remobilisation of N from protein degradation to the grain during leaf senescence. Approximately 70% of N is absorbed and assimilated before anthesis, while only 30% after anthesis. The spatial distribution of the GS isozymes may affect the NUE and the GS1/GS2 relative content/activity triggered the pool strength and influenced the N/C flow.

## Discussion

Previous studies have identified GS activity as an efficient physiological marker to indicate the N status and nutrition conditions in barley, maize and wheat^35–37^. However, no systemic study on GS isozymes in different organs during the whole growth stages of winter cultivars with different NUE was reported. Here, GS isozymes were separated using native-PAGE and the activities were assessed individually (Fig. 3c), and the transcripts GS genes and the relative subunits were also monitored in different organs from the early vegetative stage to the mature stage. It is the first time to propose GS1/GS2 relative enzyme activity or amounts, thereby providing a better understanding of the regulation of N/C metabolism in various growth stages and organs, which could influence the mechanisms involved in NUE.

Wheat N metabolism consists of absorption, assimilation, translocation, and remobilisation. The constraints on NUE was different under different N conditions; under low N, NUE was affected mainly by N absorption efficiency, while under high N levels, N assimilation and transfer efficiency had a greater impact^38^. Our study showed that the GS isozymes and N metabolic markers differed significantly between the two genotypes with different NUE from JS to 7D. A higher GS activity indicated a higher content of N metabolites, which was confirms that in higher plants and in cereals in particular^39, 40^, GS isoforms could contribute to plant biomass and yield through N metabolite assimilation and translocation. In wheat, ^15^N labelling revealed a strong correlation between GS activity and the amount of remobilised N from pre-anthesis uptake^29^. Most previous studies concentrated on the leaf of wheat, and infrequently considered the stem, glume and kernel. Considering the photosynthetic ability of the green organs, and that ^15^N labelling experiments showed that 45–60% of the ^15^N reserves assimilated before anthesis was stored in the stem and glume^41^, these organs should be examined to improve our understanding of N assimilation, translocation, and remobilisation. We detected GS isozyme activity in these organs, although the relative activity was much lower than that in the leaf. Significant differences in the N metabolites in the various organs of the two varieties were also observed, especially in the peduncle (Fig. 2).

Given the essential role in assimilating inorganic N, GS has been the focus of research for many decades^19, 42–46^. Although the activity of GS2 is generally higher than that of GS1 in C3 plant leaves, GS2 activity decreases as chloroplasts are degraded with the progression of leaf senescence^13^. In this study, the leaf and outer glume showed the same pattern, but in the peduncle and stem, GS1 activity was much higher than that of GS2 (Figs 3c and 6), suggesting that GS1 played a specific role in controlling N metabolic flux^47^. The higher relative levels of GS2 protein in the N-efficient cultivar than in the N-inefficient cultivar before anthesis suggests that GS2 plays a vital role in vegetative growth stage and carbon-nitrogen metabolic balance^24, 48^. Under N− conditions, there were higher relative levels of the GS1 protein after anthesis (Fig. 3b). The data from these N metabolism indicators (Fig. 2) and the NRE (Table S7) suggested that the N-efficient cultivar has increased assimilation ability in pre-anthesis stage and remobilisation ability in post-anthesis stage. The relative contents of the GS1 and GS2 proteins before and after anthesis could be used as a marker for breeding varieties with high NUE. A third GS isozyme activity present in the leaf and outer glume were also identified (Fig. 3c), and our previous study was mainly focus on the structure and the modification sites compared to GS2, which was regulated by the leaf development stages and it was not affected by N forms (Fig. S5). It is more likely the modified form of GS2 with different protein modification sites, but the physiological role of this new GS isoform (GS2m) remains to be elucidated^49^.

GS gene expression in the two wheat varieties in response to N supply were also analysed (Fig. 4). The significantly higher expression levels of *TaGS1* in the N-efficient cultivar compared to the N-inefficient cultivar after AS in various organs under N− conditions (Fig. 4a) likely contribute to its high protein subunits expression (Fig. 5b), which improved N translocation and remobilisation efficiency (Table S7). For example, overexpression of the *OsGS1* gene can significantly increase N metabolites, biomass, and yield, but does not constitute an overall bottleneck that limits N assimilation at the whole-plant level^42^. Avila-Ospina analysed *HvGS1* gene expression was induced by leaf senescence^30^. In the wheat leaf, the *TaGS1* gene exhibited the same pattern throughout the growth period (Fig. 4a). In the peduncle and outer glume, *TaGS1* expression was upregulated and peaked at 7D after flowering, and then decreased gradually. Under N−, although the transcript of *TaGS2* was slightly higher in the peduncle of N-efficient cultivar than that of N-inefficient cultivar, it was higher similarly in the functional leaves and outer glume of two cultivars in JS and AS (Fig. 4b). Thus, in wheat, *TaGS1* may be more important in influencing NUE after anthesis, while *TaGS2* may be more pivotal in influencing NUE before anthesis. Roots are critical for N uptake efficiency and the evidence showed that expression of GS1 genes in roots could complement soil inorganic N pools^50^. Previous studies have indicated that *OsGS1* gene expression plays a major role in carbon-nitrogen metabolism in rice later growth stage^51^. The *ZmGS2* gene is expressed mainly in the vegetative growth stage in maize^22^. Our study suggests that the *TaGS1* and *TaGS2* genes have non-redundant roles and are potential targets during specific growth development stages for enhancement of NUE.

Cellular localisation revealed the location of GS1 in the vascular and bundle sheath cells of various tissues, which is associated with a major QTL for grain protein in barley^52^. GS2 is located in the plastids of mesophyll cells in green organs, and a number of QTLs for agronomic traits related to N use and yield have been mapped to the chromosomal regions containing GS2 in wheat^53–55^. QTLs for leaf soluble protein content and chlorophyll content was also linked to a marker close to GS2 in rice^56^, ^57^. The wheat stem vascular tissue is the main channel for the circulation of total nutrients from the source to the sink, and the parenchyma of the phloem cells is the main organiser of photosynthate transportation (Figs 1e–j and 6). The development of vascular tissue is closely related to grain size, number, and filling^58, 59^, and recent studies have shown that a lack of the synthetase enzymes GS1 and GS2 in the vascular tissue resulted in reducing lignin accumulation and a disorder of the metabolic balance in rice^46^. This is the direct evidence that stem development is linked to the GS isozymes. The number and area of vascular bundles and phloem area (Table S2) may contribute to differences in the NRE (Table S7), and also affect the C/N metabolite translocation. The balance of nutriment flow in vascular tissues from the source to the sink organs would be effective to enhance NUE.

Based on the analysis of GS isozymes in the two wheat cultivars from vegetative growth to maturity^60^, phenotypic traits such as the source, sink, and flow organs (Fig. 1) are likely to be influenced by changing the regulatory processes of GS proteins^47^. According to the agronomic source-sink theory^13^ and the physiological role of the GS isozymes in regulating N metabolism, we suggest that the source and sink pool strength before or after anthesis was regulated via the relative GS1/GS2 activity or amount. The source and sink pool strength differences caused by the C-N metabolic substrate and status, leading to a flow pressure between the source and sink organs (Fig. 6), which would accelerate translocation of nutrients to the grain, and enhance the NUE when the nitrogen uptake reached a certain level^51, 61, 62^. Therefore, promoting remobilisation of the stored N from the vegetative tissues to the grain is the key to enhancing NUE. This model partly clarifies the molecular and physiological mechanisms of the GS isozymes in influencing NUE and explains the reason of the different NUE of the two genotypes. Although the mechanism presented here was at plant level, which may not extrapolated or positive related to crop level^63, 64^, it is still provides a new evidence that GS isozymes have a relationship with NUE, and it does not indicate that they directly cause NUE. More complex mechanisms should be involved in the larger C-N metabolism interacting networks, such as transcription factors, hormones and other refined regulatory mechanisms, and how they interact with environmental conditions^65, 66^.

## Materials and Methods

### Pots Experimental Design and Sampling

The experiment was conducted in pots during the 2013–2014 growing season at the experimental station of Henan Agricultural University in Zhengzhou, Henan Province, PRC (113°59′E, 34°86′N, 110 m ASL). The soil (sandy loam) was obtained from a field where the previous crop had been *Z. mays* L. A total of 20 kg of soil (air dried weight, sieved through a 0.8-cm mesh) mixed with fertiliser was packed into pots (35-cm height, 38-cm diameter) at a density of 1.12 g cm^−1^. The sieved soil contained 0.79 g total N kg^−1^, 68.50 mg available N kg^−1^, 14.35 mg available phosphate kg^−1^, 143.89 mg available potassium kg^−1^, and 11.31 g organic matter kg^−1^ with a pH of 7.90. The experiment used a criss-cross design with three N levels (N+, 3.02 g urea pot^−1^; N−, 1.61 g urea pot^−1^; N0, 0 g urea pot^−1^, and analytically pure) and two winter wheat cultivars, YM49 (N-efficient) and XN509 (N-inefficient) (Fig. S1). The fertiliser contained 1.51 g urea pot^−1^ (N+), 0.81 g urea pot^−1^ (N−), 0 g urea pot^−1^ (N0), 2.86 g calcium superphosphate pot^−1^, 2.75 g potassium sulphate pot^−1^ (analytically pure), and the remainder of the urea was dissolved in water and applied at the jointing stage. Soil was maintained at 70% of water holding capacity. The two typical wheat cultivars (N-efficient and N-inefficient) were screened out from sixteen cultivars in two growing seasons (Fig. S1), and sown on 14th October 2013, 15 seeds were sown in each pot. The meteorological data of growth seasons on air temperature, rainfall, relative humidity and solar radiation were acquired from device (Watch Dog 2900ET, USA) installed next to the field (Table S3).

Plant material sampled for the physiological indicators (N and C metabolite contents and GS enzyme activity) and gene expression studies was based on the Feekes scale at Feekes 3.0 (wintering stage, WS), Feekes 5.0 (jointing stage, JS), Feekes 10.5.1 (anthesis stage, AS), Feekes 11.1 (7 days after anthesis, 7D), Feekes 11.2 (14 days after anthesis, 14D), Feekes 11.3 (21 days after anthesis, 21D), and Feekes 11.4 (maturity stage, MS), sampling date of two genotypes was provided in Table S4. The main stem and the other tillers were separated and divided into functional leaves (for WS and JS, this was the largest expanding leaf, and after anthesis, this was the flag leaf), peduncle, outer glume, kernel and root. Some of the tissues were frozen in liquid N for 3 h and then stored at −80 °C until further analysis. The remaining tissues were dried at 80 °C to determine the dry weight and total N content.

### Enzymatic Assays and Metabolite Analysis

#### GS Enzymatic Activity

GS activity was measured according to the method of O’Neal and Joy^67^.

#### Chlorophyll Content

Chlorophyll content was measured using the alcohol and acetone extraction, spectrophotometer colorimetric method^68^.

#### Total N Content and Accumulation

Tissues were crushed and total N content was measured using the semi-micro Kjeldahl method where total N accumulation = dry matter weight of tissues × total N content^68^.

#### Free Amino Acid Content

Free amino acid content was measured using colorimetry analysis^68^.

#### Soluble Protein

Soluble protein was determined using the Coomassie brilliant blue G250 staining method^68^.

#### Soluble Sugar

Soluble sugar was determined using the anthrone colorimetry method^68^.

### In-gel Detection of GS Activity and Relative Activity Analysis

Gel systems (at 4 °C) were used to separate the proteins and detect GS1 and GS2 isozymes activity. The native gel system used a 1.5 × 170 × 100-mm gel. The resolving gel was 5% polyacrylamide (pH 8.7), and the stacking gel was 3% polyacrylamide (pH 6.7). Samples were normalised to 50 μL from 0.5 g fresh weight (FW) of organs in each lane, and electrophoresis was carried out at 80 V for the stacking gel and 120 V for the resolving gel.

After electrophoresis, GS activity was detected in-gel by the conversion of l-glutamine to γ-glutamyl hydroxamate^69^. The gel was immersed in 100 mL of reaction buffer (100 mM Tricine, 1.3 mM EDTA, 20 mM sodium arsenate, 20 mM MgSO_4_, 0.5 mM ADP, 25 mM hydroxylamine, and 50 mM l-glutamate, pH 7.4) and incubated at 37 °C for 30 min with slow shaking, after which the reaction buffer was removed. The reaction was terminated by the addition of 50 mL of stop solution (370 mM FeCl_3_, 200 mM trichloroacetic acid, and 700 mM HCl) for 3 min until GS activity appeared as a brownish band on the yellow background. The gel was washed twice with cool distilled H_2_O and scanned immediately. The amount of protein was estimated by grey scanning using Gel-Pro analyser software (version 4.0 for Windows, Media Cybernetics, Rockville, MD, USA).

### RNA Extraction and cDNA Synthesis

Total RNA was isolated from the functional leaf, peduncle, and outer glume of individual plants of the two wheat cultivars grown under various N levels, using RNAiso Plus extraction reagent (Takara, Tokyo, Japan). The RNA quality was confirmed with a 1% agarose gel and quantified with a spectrophotometer. Three replicates were carried out for RNA isolation. Total RNA from each replicate (2 μg) and Hi-Script Reverse Transcriptase with gDNA Wiper Mix (Vazyme Biotech, Nanjing, China) was used to prepare cDNA. The conditions for a 20 μL-reaction were as follows: 2 μL total RNA, 5 μL 4 × gDNA Wiper Mix, and 9 μL RNase-free H_2_O at 42 °C for 2 min to digest the DNA, followed by the addition of 4 μL 5 × SuperMixII and incubation at 25 °C for 10 min, 42 °C for 30 min, with a final denaturation step at 85 °C for 5 min.

### Primer Design and Quantitative Real-Time PCR Analysis

The mRNA sequences of the wheat GS1 and GS2 genes were obtained from the National Center for Biotechnology Information database (NCBI, GenBank accession numbers: HQ840647 and JF894116) and gene-specific primers were designed using Primer Premier 5.0 software (Table S1). RT-PCR was performed using individual cDNAs obtained from each of the three replicates from the pot experiments, to generate GS1 and GS2 expression profiles. RT-PCR reactions were performed using HiScript II Q RT SuperMix for quantitative PCR (Vazyme Biotech) in a Bio-Rad iQ5 RT-PCR thermal cycler (Bio-Rad, Hercules, CA, USA). The reverse transcription efficiencies of the GS1, GS2, and GAPDH genes were almost the same when the Ct values at different dilutions of cDNA were analysed^70^. The reaction was performed using 1 μL of cDNA, 15 pmol of gene-specific primer, and 25 μL of 2 × Q RT SuperMix for a qPCR in a 50-μL reaction volume. The following amplification program was used: 95 °C for 7 min, 40 cycles at 95 °C for 10 s, primer annealing at 58 °C for 30 s, 72 °C for 15 s, 58 °C for 10 s, and 95 °C for 15 s. All samples were amplified in triplicate, and the mean and standard error values were calculated. A completely randomised design was used to analyse the RT-PCR data.

### GS Subunit Identification

Proteins were extracted at 4 °C in 25 mM Tris-HCl, pH 7.5, 0.5 mM EDTA, and denatured at 100 °C for 5 min after the addition of one volume of 2 × SDS buffer (v/v) to one volume of protein extract. Equal amounts of protein were loaded in each lane and separated by SDS-PAGE on 12.5% polyacrylamide gels. Electrophoresis was carried out at 80 V for the stacking gel and 120 V for the resolving gel. Denatured proteins were transferred to PVDF membrane using the Trans-Blot Turbo transfer system (Bio-Rad) at 30 V for 20 min. For the GS blotting, polyclonal antibodies were used to detect both the GS1 and GS2 isozymes.

### Scanning Electron Microscopy (SEM) Observation and Analysis

The main stem at the base of the second internode of the two cultivars was examined at 7 d after flowering under low N. Stem segments (~2 mm) from the base were removed and placed in 4% glutaraldehyde fixative for 4 h, and washed three times for 10 min in 0.1 M phosphate buffer solution (pH 7.2). Samples were then fixed in osmium tetroxide for 2 h, and washed three times for 10 min in 0.1 M phosphate buffer. The sections were dehydrated sequentially in ethanol at concentrations of 50, 50, 70, and 100% for 10 min. Critical point drying with CO_2_ or freeze-drying was performed. Samples were kept in a desiccator until metallisation. An FEI Quanta 200 scanning electron microscope (FEI Company, Eindhoven, Netherlands) at 10e15 kV was used for the SEM observations and analysis.

## Electronic supplementary material

Dataset 1
