# Supplementary material for: The role of glutamine synthetase isozymes in enhancing nitrogen use efficiency of N-efficient winter wheat
Source: Sci Rep. 2017 Apr 20;7:1000. doi: 10.1038/s41598-017-01071-1 (PMC5430530; doi:10.1038/s41598-017-01071-1)
Supplement: Supplementary file 1 — Dataset 1 [file 41598_2017_1071_MOESM1_ESM.doc]

New perspective on the role of glutamine synthetase isozymes in enhancing nitrogen use efficiency of winter wheat

Zhiyong Zhang^1,2,3^, Shuping XIONG^1,2,3^, Yihao WEI^1,2^, Xiaodan Meng^1,2,3^, Xiaochun WANG^4^ and Xinming MA^1,2,3^

Supplementary date

Supplemental Table S1 List of gene-specific primers used to determine wheat GS1 and GS2 gene expression profiles.

Supplemental Table S2 Analysis of the stem structural characteristics of the two wheat varieties under N– conditions.

Supplemental Table S3 Meteorological data of growth seasons on air temperature, rainfall, solar radiation and relative humidity.

Supplemental Table S4 Sampling date and phenological periods with Feekes scales of two genotypes.

Supplemental Table S5 Datasets of grains and yield components of two genotypes.

Supplemental Table S6 Nitrogen use efficiency (NUE) of two wheat genotypes over two seasons.

Supplemental Table S7 Nitrogen remobilisation efficiency and N contribution rate to the grains of two varieties under N– and N+ conditions.

Supplemental Fig. S1 Nitrogen physiological efficiency of sixteen wheat cultivars in 2012-2013 and 2013-2014

Supplemental Fig. S2 Datasets of Leaf morphology and photosynthetic ability.

Supplemental Fig. S3 Grain filling rate and dry matter accumulation of two wheat genotypes under various N conditions

Supplemental Fig. S4 GS isozymes in the leaf and kernel of sixteen wheat genotypes.

Supplemental Fig. S5 Effects of N forms to GS isozymes in leaves of different positions.

Supplemental Fig. S6. GS isozymes in kernel of two wheat genotypes were separated by native-PAGE.

Table S1. List of gene-specific primers used to determine wheat GS1 and GS2 gene expression profiles.

| Gene name | Forward primer sequence (5'–3') | Reverse primer sequence (5'–3') | Amplicon size (bp) |
| --- | --- | --- | --- |
| *TaGS1* | CCGACTGTTGGCATTTCT | TGTAGTTTGTGTGAGCACCAG | 148 |
| *TaGS2* | GAAATCAGTGGAACAAACGG | CTCCCGCATCAATACCAAC | 82 |
| *TaGAPDH* | CCAGAAGACTGTTGATGGTCC | AGTGCTGCTTGGAATGATGT | 82 |

The mRNA sequences of wheat GS1 and GS2 genes were obtained from the National Centre for Biotechnology Information (NCBI, GeneBank accession numbers: HQ840647 and JF894116) and gene-specific primers were designed using Primer Premier 5.0 software.

Table S2. Analysis of the stem structural characteristics of the two wheat genotypes under N– conditions

| Wheat  Genotype | Thickness of  stem wall (μm) | Vascular bundle number (per stem) | | | Vascular bundle area  (μm^2^) | | | Phloem area  (μm^2^) | | |
| --- | --- | --- | --- | --- | --- | --- | --- | --- | --- | --- |
|  |  | Inner | Middle | Outer | Inner | Middle | Outer | Inner | Middle | Outer |
| YM49 | 1244 | 43 | 7 | 33 | 26481 | 16470 | 5875 | 2214 | 809 | 415 |
| XN509 | 904 | 38 | 9 | 25 | 23122 | 15124 | 3364 | 1661 | 932 | 372 |

Table S3. Meteorological data of growth seasons on air temperature, rainfall, solar radiation and relative humidity

| Month | Average air temperature | Rainfall | Daily solar radiation | Relative humidity |
| --- | --- | --- | --- | --- |
|  | (℃) | (mm) | (kWh/m^2^/d) | (%) |
| Oct-2013 | 17.15 | 26.30 | 3.29 | 52.80 |
| Nov-2013 | 9.66 | 32.50 | 2.85 | 53.70 |
| Dec-2013 | 3.59 | 0.02 | 2.61 | 53.80 |
| Jan-2014 | 4.04 | 0.11 | 2.88 | 53.70 |
| Feb-2014 | 2.64 | 24.30 | 3.43 | 52.40 |
| Mar-2014 | 12.99 | 6.90 | 4.20 | 52.20 |
| Apr-2014 | 17.09 | 56.40 | 5.10 | 46.10 |
| May-2014 | 24.12 | 57.60 | 5.38 | 49.10 |

Data was downloaded from Watch Dog 2900ET, USA.

Table S4. Sampling date and phenological periods with Feekes scales of two wheat genotypes

| Sampling stages | | Sampling Date | | | |
| --- | --- | --- | --- | --- | --- |
| Phenological period | Feekes Scale | YM49 | | XN509 | |
|  |  | N- | N+ | N- | N+ |
| Sowing date | — | Oct. 14th, 2013 | Oct. 14th, 2013 | Oct. 14th, 2013 | Oct. 14th, 2013 |
| WS | Feekes 3.0 | Jan. 15th, 2014 | Jan. 15th, 2014 | Jan. 15th, 2014 | Jan. 15th, 2014 |
| JS | Feekes 5.0 | Mar. 17th, 2014 | Mar. 18th, 2014 | Mar. 14th, 2014 | Mar. 16th, 2014 |
| AS | Feekes 10.5.1 | Apr. 18th, 2014 | Apr. 20th, 2014 | Apr. 16th, 2014 | Apr. 18th, 2014 |
| 7D | Feekes 11.1 | Apr. 25th,2014 | Apr. 27th, 2014 | Apr. 23th, 2014 | Apr. 25th, 2014 |
| 14D | Feekes 11.2 | May 2th, 2014 | May 4th, 2014 | Apr. 30th, 2014 | May 2th, 2014 |
| 21D | Feekes 11.3 | May 9th, 2014 | May 11th, 2014 | May 7th, 2014 | May 9th, 2014 |
| MS | Feekes 11.4 | May 24th, 2014 | May 27th, 2014 | May 20th, 2014 | May 24th, 2014 |

Table S5. Datasets of grains and yield components of two wheat genotypes

| Wheat  genotype | N level | Ear number per plant | Grain number per ear | 1000-grain weight (g) | Yield per plant (g) |
| --- | --- | --- | --- | --- | --- |
| YM49 | N0 | 2.30±0.24 b | 32.3±0.87 c | 47.47±0.89 a | 6.05±0.45 b |
|  | N- | 3.51±0.35 a | 36.1±1.32 ab | 48.46±1.21 a | 7.76±1.25 a |
|  | N+ | 3.58±0.37 a | 37.2±0.95 a | 48.31±1.11 a | 7.79±1.09 a |
| XN509 | N0 | 2.31±0.12 b | 32.8±1.21 c | 40.03±0.98 b | 6.59±0.25 b |
|  | N- | 3.46±0.28 a | 35.6±1.10 b | 40.42±0.75 b | 6.49±0.42 b |
|  | N+ | 3.76±0.19 a | 38.7±0.98 a | 40.86±0.87 b | 7.11±0.29 a |

SE indicates standard error of the mean. Letters indicate significant differences between treatments within one year (α = 0.05, Fisher’s LSD).

Table S6. Nitrogen use efficiency (NUE) of two wheat genotypes over two seasons.

| Wheat genotype | N level | NUE (kg kg^–1^ ± SE) | | CV(%) |
| --- | --- | --- | --- | --- |
|  |  | 2012–2013 season | 2013–2014 season |  |
| YM49 | N– | 31.04 ± 0.65 a | 30.39 ± 0.49 a | 2.93 |
|  | N+ | 30.12 ± 1.44 a | 28.81 ± 1.14 a | 4.63 |
| XN509 | N– | 17.15 ± 0.78 b | 20.86 ± 1.09 b | 8.50 |
|  | N+ | 14.91 ± 0.61 c | 15.87 ± 0.77 c | 5.29 |

SE indicates standard error of the mean. Letters indicate significant differences between treatments within one year (α = 0.05, Fisher’s LSD). CV (coefficient of variation).

Table S7. Nitrogen remobilisation efficiency and N contribution rate to the grains of two varieties under N– and N+ conditions.

| Wheat genotype | N  level | N Accumulation (mg) | | | Pre-NT (mg) | Post-NA (mg) | NRE (%) | Pre-NCR (%) | Post-NCR (%) |
| --- | --- | --- | --- | --- | --- | --- | --- | --- | --- |
|  |  | Anthesis | Maturity | Grain |  |  |  |  |  |
| YM49 | N– | 108.37 b | 166.98 a | 139.71 a | 81.11 b | 58.60 a | 74.84 a | 58.06 d | 41.94 a |
|  | N+ | 151.85 a | 164.28 a | 127.71 a | 115.29 a | 12.43 c | 75.92 a | 90.27 a | 9.73 d |
| XN509 | N– | 77.52 c | 102.54 c | 79.48 c | 54.46 c | 25.02 b | 70.26 b | 68.52 c | 31.48 b |
|  | N+ | 108.59 b | 124.37 b | 97.09 b | 81.31 b | 15.78 c | 74.87 a | 83.75 ab | 16.25 cd |

A single plant was sampled from every pot treatment and evaluated for total N (five biological replicates). LSD value is for *p* < 0.05. Pre-NT (pre-anthesis N translocation) = NA_anthesis_ – (NA_maturity_ – NA_grain_); Post-NA (post-anthesis N Accumulation) = NA_maturity_ – NA_anthesis_; NRE (N remobilisation efficiency) = [NA_anthesis_ – (NA_maturity_ – NA_grain_)] / NA_anthesis_ × 100; Pre-NCR (pre-anthesis N contribution rate to grain) = [NA_grain_ – (NA_maturity_ – NA_anthesis_)] / NA_grain_ × 100; Post-NCR (post-anthesis N contribution rate to grain) = (NA_maturity_ – NA_anthesis_) / NA_grain_ × 100.

Fig. S1 Nitrogen physiological efficiency of sixteen wheat cultivars in 2012-2013 and 2013-2014


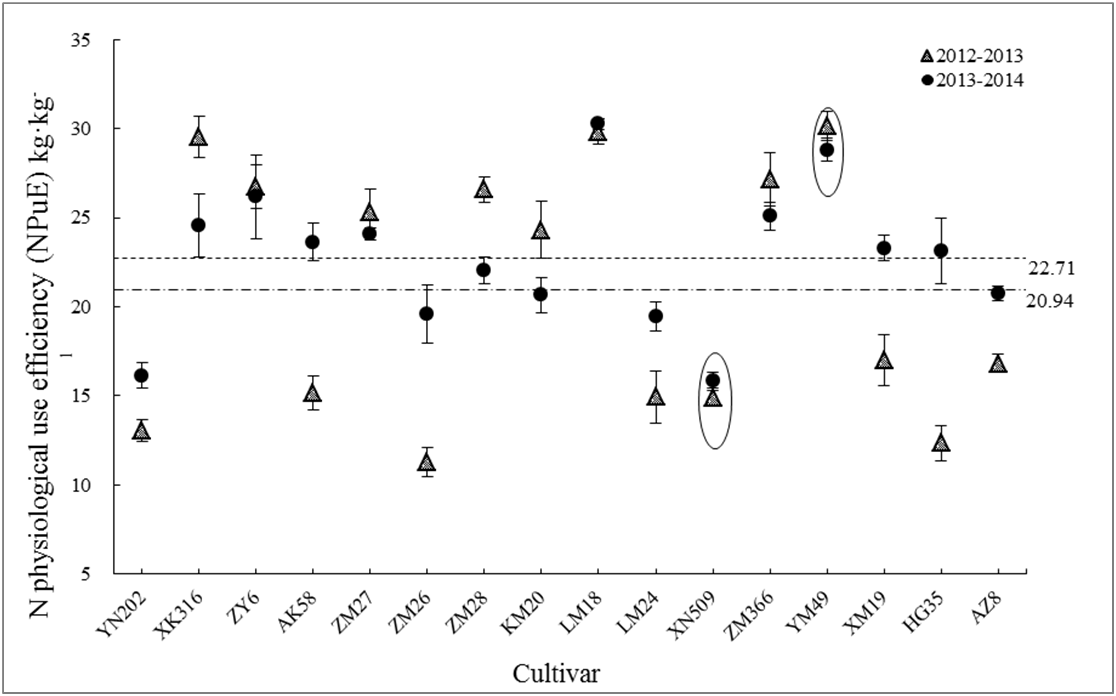


Fig. S2 Datasets of leaf morphology and photosynthetic ability.


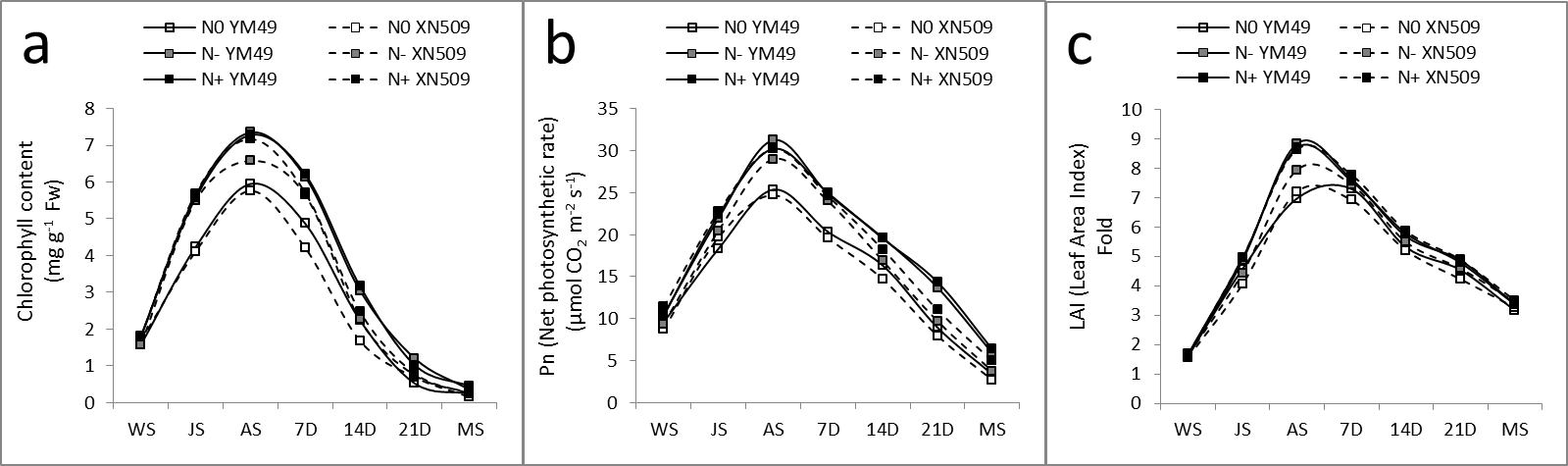
a. Chlorophyll content of functional leaves over a time-course. b. Net photosynthetic rate of functional leaves over a time-course. c. Leaf area index over a time-course.

Fig. S3 Grain filling rate and dry matter accumulation of two wheat genotypes under various N conditions.


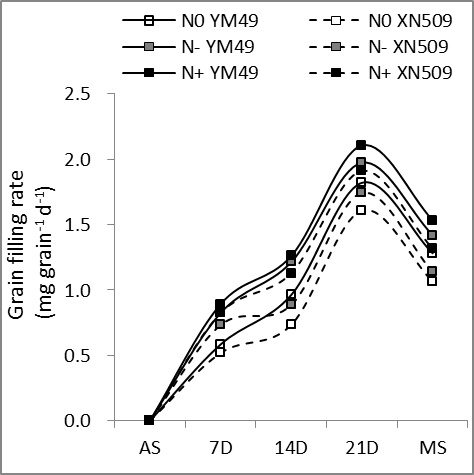

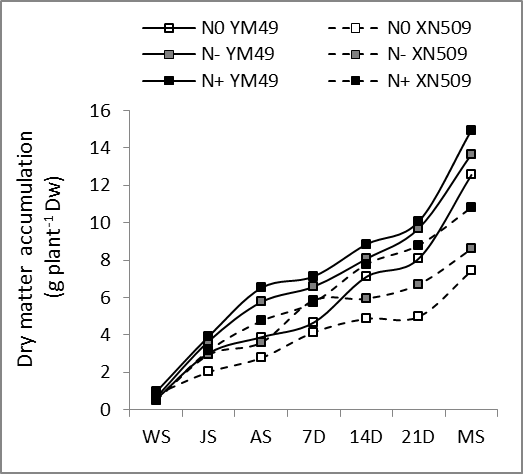


a

b

a. Grain filling rate of two cultivars under various N conditions. b. Dry matter accumulation of two cultivars under various N conditions. Data was collected from single plant with five replicates.

Fig. S4. GS isozymes in the leaf and kernel of sixteen wheat genotypes.


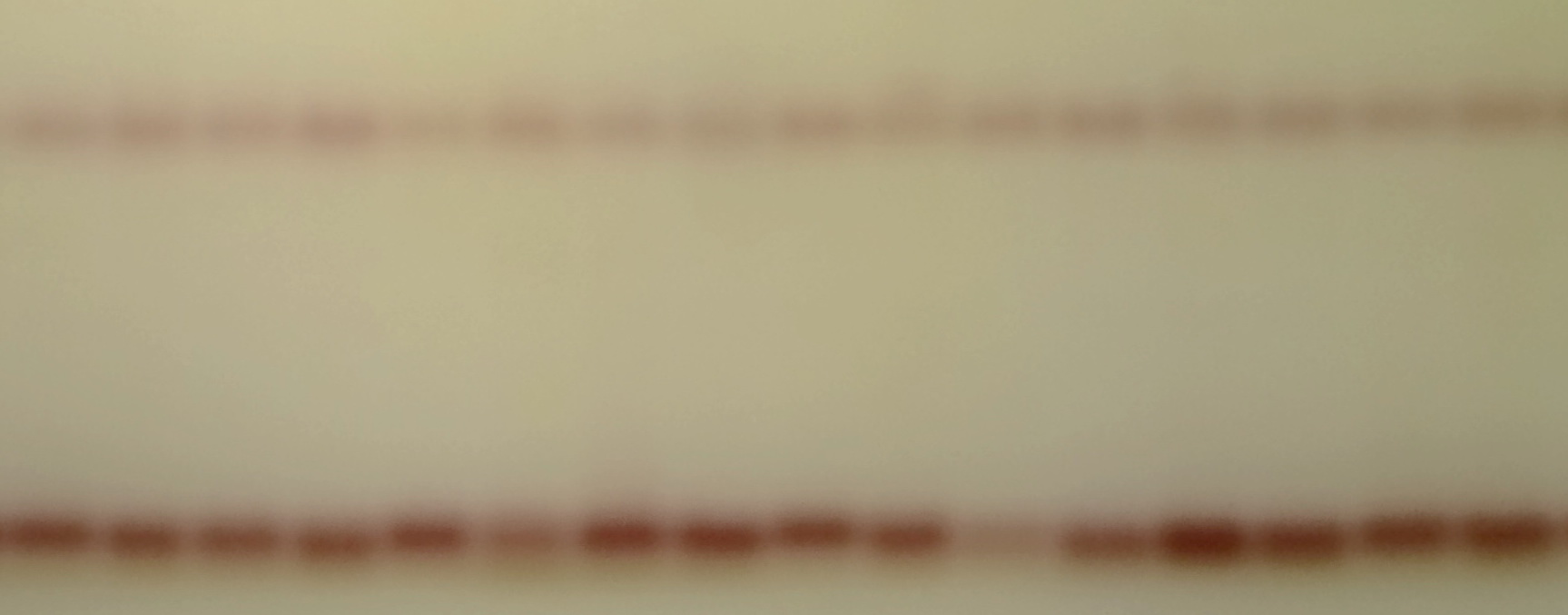


GS1

YN202

XK316

ZY6

AK58

ZM27

ZM26

ZM28

KM20

LM18

LM24

XN509

ZM366

YM49

XN19

HG35

AZ8

GS2


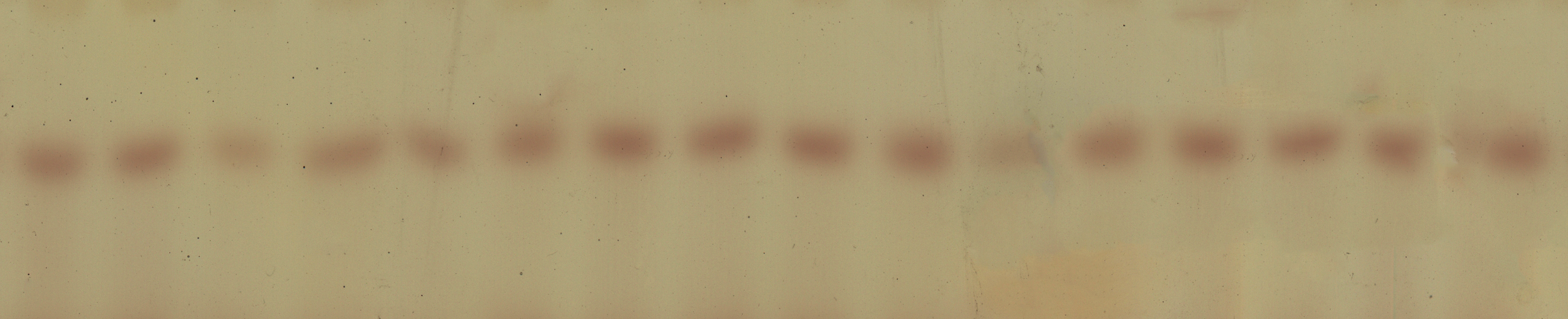


GS1

YN202

XK316

ZY6

AK58

ZM27

ZM26

ZM28

KM20

LM18

LM24

XN509

ZM366

YM49

XN19

HG35

AZ8

a

b

a. GS isozymes in the leaf of sixteen wheat cultivars; b. GS isozymes in the kernel of sixteen wheat cultivars. Note: Samples were taken at jointing stage. GS proteins were separate using native-PAGE (5%) with a transferase assay.

Fig. S5 Effects of N forms to GS isozymes in leaves of different positions.


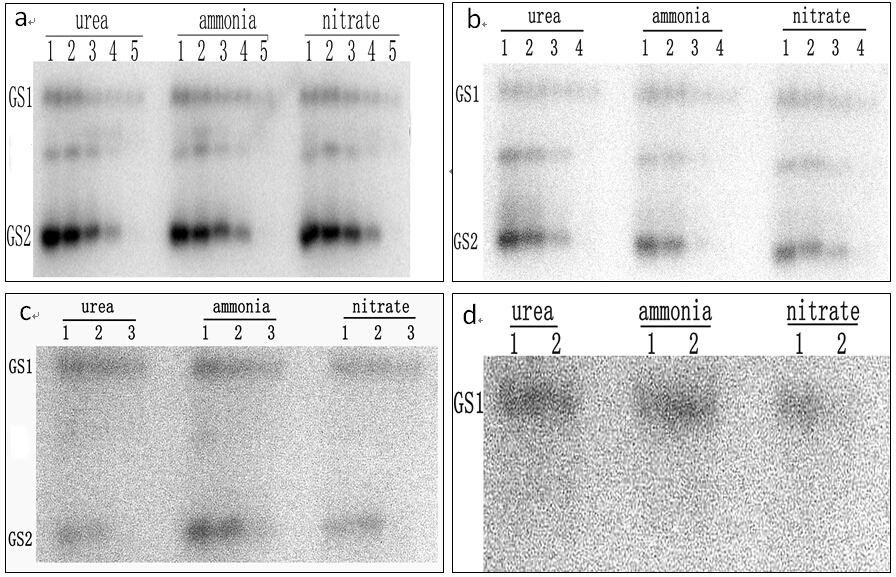


GS2m

GS2m

Note: Cultivar: YM49, a: AS; b: 7D; c: 14D; d: 21D; 1: Flag leaf; 2: The second leaf; 3: The third leaf; 4: The forth leaf; 5: The fifth leaf.

Fig. S6 GS isozymes in kernel of two wheat genotypes were separated by native-PAGE.


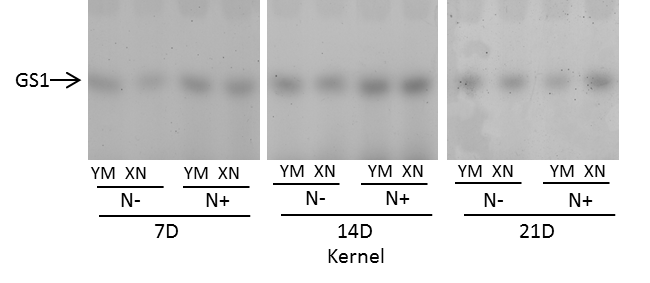


Note: Equal protein amounts were loaded in each lane (50 μL)
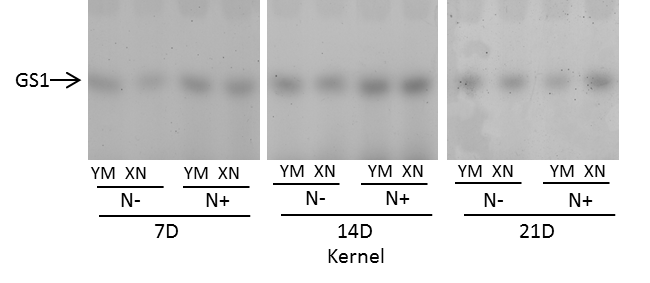
.
